# Characterization of transcriptional landscape in bone marrow-derived mesenchymal stromal cells treated with aspirin by RNA-seq

**DOI:** 10.7717/peerj.12819

**Published:** 2022-01-24

**Authors:** Xinpeng Liu, Yuanbo Zhan, Wenxia Xu, Lixue Liu, Xiaoyao Liu, Junlong Da, Kai Zhang, Xinjian Zhang, Jianqun Wang, Ziqi Liu, Han Jin, Bin Zhang, Ying Li

**Affiliations:** 1 Heilongjiang Provincial Key Laboratory of Hard Tissue Development and Regeneration, The Second Affiliated Hospital of Harbin Medical University, Harbin, China; 2 The Second Affiliated Hospital of Harbin Medical University, Department of Periodontology and Oral Mucosa, Harbin, China; 3 Heilongjiang Academy of Medical Sciences, Harbin, China

**Keywords:** Aspirin, RNA-seq, Cell senescence, Immune response, Lipid metabolism, BM-MSCs

## Abstract

**Introduction:**

Aspirin is a common antipyretic, analgesic, and anti-inflammatory drug, which has been reported to extend life in animal models and application in the treatment of aging-related diseases. However, it remains unclear about the effects of aspirin on bone marrow-derived mesenchymal stromal cells (BM-MSCs). Here, we aimed to analyze the influence of aspirin on senescence and young BM-MSCs.

**Methods:**

BM-MSCs were serially passaged to construct a replicative senescence model. SA-β-gal staining, PCR, western blot, and RNA-sequencing were performed on BM-MSCs with or without aspirin treatment, to examine aspirin’s impact on bone marrow-derived mesenchymal stem cells.

**Results:**

SA-β-gal staining, PCR, and western blot revealed that aspirin could alleviate the cellular expression of senescence-related indicators of BM-MSCs, including a decrease of SA-β-gal-positive cells and staining intensity, and downregulation of p16, p21, and p53 expression after aspirin treatment. RNA-sequencing results shown in the biological processes related to aging, aspirin could influence cellular immune response and lipid metabolism.

**Conclusion:**

The efficacy of aspirin for retarding senescence of BM-MSCs was demonstrated. Our study indicated that the mechanisms of this delay might involve influencing immune response and lipid metabolism.

## Introduction

Tissue defects and functional impairment can be compensated by the coordination between differentiation and proliferation of specific stem cells or progenitors. Bone marrow-derived mesenchymal stromal cells (BM-MSCs) generated from mesoderm, one of the most well-characterized stem cells, possess self-renewal ability and multi-directional differentiation potential. They can differentiate into multiple lineages such as osteocytes, adipocytes, chondrocytes, neural cells, myocytes, and stromal cells supporting hematopoiesis (Pittenger et al., 1999). Moreover, BM-MSCs possess immunosuppressive activity (Petrie Aronin & Tuan, 2010), which may benefit immunomodulation and tissue repair. Pioneering studies have shown that BM-MSCs can generate therapeutic results in bone defect (Warnke et al., 2004), skin regeneration (Yang et al., 2011), autoimmune diseases (Parekkadan, Tilles & Yarmush, 2008), and cardiovascular disease (Forrester, Price & Makkar, 2003). However, what is limiting is infrequent BM-MSCs obtained from the recipient’s bone marrow and restricted expansion capability accompanied by replicative senescence (Ren et al., 2013; Wagner, Ho & Zenke, 2010). This replicative senescence of BM-MSCs not only causes the cells to lose normal morphology but the functional decline also contributes to impairments in proliferation, differentiation, and stemness levels (Galderisi et al., 2009; Sethe, Scutt & Stolzing, 2006; Zheng et al., 2016). For clinical therapeutic applications, overcoming cellular replicative senescence of BM-MSCs remain to be delineated.

Aspirin is one of the world’s most widely used drugs. It is an oral antithrombotic agent for cardiovascular disease and in the treatment of chronic rheumatic diseases based on its anti-inflammatory, and anti-rheumatic activities. Nevertheless, the potential and real benefits of aspirin therapy go beyond its regular usage, including prevention and treatment of cancer (Patrignani & Patrono, 2018), reduced incidence of postmenopausal osteoporosis (Ma, Liu & Sun, 2019), and, in animal models, extended lifespan, an effect seen in *Caenorhabditis elegans* (Wan et al., 2013), *Drosophila melanogaster* (Danilov et al., 2015), and *Mus musculus* (Strong et al., 2008). This could happen through several mechanisms; for example, aspirin interferes with cytokine response processes, oxidant production, and blocking glycoxidation reactions that can improve lifespan in long-lived species by antioxidant therapies (Phillips & Leeuwenburgh, 2004), and inhibition of cyclooxygenase I (COX I) and cyclooxygenase II (COX II), which results in downregulation of prostaglandins to reduce the inflammatory response (Weissmann, 1991).

To date, no research has concentrated on whether aspirin can delay the senescence of BM-MSCs by counteracting cellular inflammatory responses. In this present study, we aimed to analyze aspirin’s effects on early passages (EP) BM-MSCs and late passages (LP) BM-MSCs.

## Materials and Methods

### Cell culture and treatment of bone marrow-derived mesenchymal stromal cells

Commercial Sprague Dawley rat BM-MSCs (RASMX-01001; Cyagen Biosciences, Guangzhou, China) were used for all the experiments. BM-MSCs were cultured at 37 °C and 5% CO_2_ in SD Rat Mesenchymal Stem Cell Growth Medium (RASMX-90011; Cyagen Biosciences, Guangzhou, China), which contained basal medium, 10% fetal bovine serum, 1% penicillin-streptomycin, and 1% glutamine. Passage 3~5 (young) (EP) and passage 15~20 (aging) (LP) BM-MSCs were cultured until confluence reached 70~90%. EP and LP cells were treated with 400 μmol/L aspirin (A2093; Sigma-Aldrich, St. Louis, MO, USA) for 48 h. This dosage application used here was selected from our previous studies showing that it did not affect the apoptosis and proliferation of BM-MSCs (Zhan et al., 2018). Untreated cells were used as controls.

### Senescence-associated β-galactosidase (SA-β-gal) staining

Cellular senescence was determined by SA-β-gal staining (C0602; Beyotime Biotech., Jiangsu, China). Briefly, cells were seeded in a 6-well plate (Thermo, Waltham, MA, USA) at a density of 2 × 10^4^/well. After 24 h, EP and LP BM-MSCs were treated with 400 μmol/L aspirin for 48 h, respectively. Subsequently, cells were fixed with 4% paraformaldehyde for 15 min at room temperature and washed three times with PBS. PBS was replaced with SA-β-gal staining solution, the plate sealed with plastic wrap at 37 °C, and incubated overnight. The following day, cells were washed with PBS three times and then observed by light microscope in five random fields of view. The experiment was repeated three times. The percentage of SA-β-gal positive cells was calculated by counting the number of blue cells at least 100 cells per sample.

### Quantitative real-time PCR verification of senescence-associated gene

With the intent to explore aging-related gene expression, real-time PCR was performed. Total RNA was extracted from cells using TRIzol (TaKaRa, Kusatsu, Japan) in terms of the manufacturer’s protocol. The extracted RNA was reverse transcripted into cDNA with the PrimeScript RT reagent kit (TaKaRa, Kusatsu, Japan). Subsequently, quantitative real-time PCR experiments were performed using the MxPro-Mx3000P real-time PCR System (Stratagene, La Jolla, CA, USA) based on SYBR Green I fluorescence. The reaction was performed in 20 μl using SYBR Premix Ex TaqTM kit (TaKaRa, Kusatsu, Japan) using the manufacturer’s protocol. The condition was set for 2 min at 95 °C, followed by 40 cycles of 95 °C for 15 s, 60 °C for 30 s, 72 °C for 30 s, accompanied with 95 °C for 10 s, 60 °C for 5 s and 95 °C for 5 s. For real-time PCR the sequences of the following primers were used: p53: 5′-TGA CTT TAG GGC TTG TTA TGA GAG–3′ (Forward), 5′-CAG CAG AGA CCC AGC AAC TAC–3′ (Reverse); p16: 5′-GAT GGG CAA CGT CAA AGT GG–3′ (Forward), 5′-TAC CGC AAA TAC CGC ACG AC–3′ (Reverse); p21: 5′-GGG AGG GCT TTC TTT GTG TA–3′ (Forward), 5′-GCA TCG TCA ACA CCC TGT CT–3′ (Reverse); GAPDH: 5′-GACAACTCCCTCAAGATTGTCAG–3′ (Forward), 5′-ATG GCA TGG ACT GTG GTC ATG AG–3′ (Reverse). Relative gene expression was calculated by the 2^−ΔΔ^Ct method, and the level relative to GAPDH was normalized.

### Western blot

Cells were collected and protein lysates were prepared with RIPA lysis buffer (Beyotime, Shanghai, China). Protein concentration was quantified with BCA Protein Assay Kit (Beyotime, Shanghai, China). Forty micrograms (μg) of protein lysates were split into 12% sodium dodecyl sulfate-polyacrylamide gel electrophoresis (SDS-PAGE) gels and transferred to polyvinylidene difluoride (PVDF) membranes (Beyotime, Shanghai, China). Followed by blocking in 5% non-fat milk for 1 h at room temperature, the immunoblots were examined overnight 4 °C with the under antibodies: anti-p21 (Cat. No. ab109199, 1:1,000; Abcam, Cambridge, UK), anti-p53 (Cat No. 10442-1-AP, 1:1,000; Proteintech, Rosemont, IL, USA) and anti-β-actin (Cat. No. 8457S, 1:1,000; Cell Signaling Technology, Inc., Danvers, MA, USA). For secondary antibodies, peroxidase-conjugated IgG (#BL003A, 1:5,000, Biosharp, Hefei, China) was used, followed by chemiluminescence detection with an enhanced chemiluminescence kit (Biosharp, Hefei, China).

### RNA sequencing

Total RNA extraction was done from BM-MSCs after different treatments with TRIzol reagent. Subsequently, the RNA samples were sent to BGI Co., LTD (Shenzhen, China), and BGISEQ-500 platform was applied to perform RNA-sequencing. Differentially expressed genes were determined based on Q value (Adjusted *P*-value) <= 0.05 (Love, Huber & Anders, 2014).

### Analysis of functional and pathway enrichment

Biological function Gene Ontology (GO), Kyoto Gene and Genome Encyclopedia (KEGG) signal pathway, and gene set enrichment analysis (GSEA) enrichment analysis were performed in the Dr. Tom online software. In the GSEA analysis, normalized enrichment score (NES) ≥ 1 or ≤−1, nominal *P*-value (NOM *P*-val) <0.05, and false discovery rate (FDR q-val) <0.25 were considered as the cutoff values.

### Gene set variation analysis

With the intent to explore pathway activity in the 50 hallmark pathways, we utilized gene set variation analysis (GSVA) analysis. GSVA analysis was capable of identifying pathways with significant differences in diverse treatment groups, which were more biologically far-reaching than genetic analysis (Hänzelmann, Castelo & Guinney, 2013).

### Statistical analysis

Throughout the text, we performed an unpaired Student’s t-test, and a *P* value < 0.05 was examined statistically significant. Each experiment was repeated at least three times. GraphPad Prism 7.0 was utilized for statistical analysis.

## Results

### Aspirin treatment reduces cellular senescence in bone marrow-derived mesenchymal stromal cells

**Figure 1:**
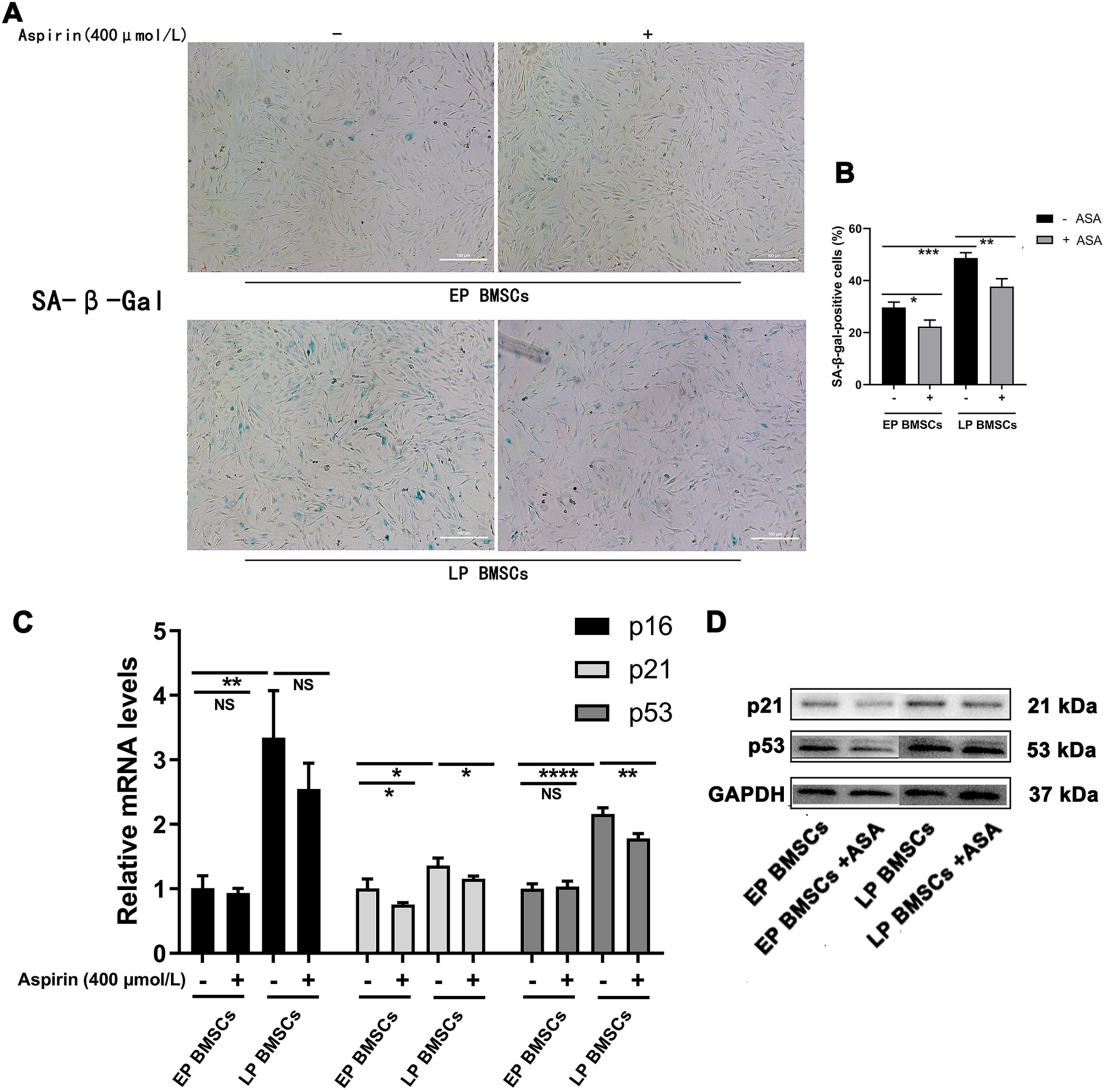
Aspirin treatment reduces replicative senescence of BM-MSCs. (A) Senescence-associated β-galactosidase (SA-β-gal) staining of EP and LP BM-MSCs with or without aspirin treatment (400 μmol/L). Scale bar = 100 μm. (B) Percentages of cells that showed positive activity of SA-β-gal after each treatment scheme. At least 100 cells were examined in each of the three replicate experiments for different groups. *P < 0.05, **P < 0.01, ***P < 0.001. (C) Gene expression of p16, p21, and p53 in different groups. *P < 0.05, **P < 0.01, ****P < 0.0001, NS: no significance, with comparisons indicated by lines. (D) Western blot of p21 and p53 in EP and LP BM-MSCs with or without aspirin exposure, GAPDH served as an internal control.

We began by performing SA-β-gal staining in BM-MSCs with or without aspirin treatment at early and late passages. To explore the potential relationship between aspirin treatment and senescence, we examined the SA-β-gal staining, which was optimally active at a pH of 6.0 (Figs. 1A, 1B). The percentage of SA-β-gal-positive cells in EP BM-MSCs populations was initially low. In contrast, almost all LP BM-MSCs were SA-β-gal positive (*P* < 0.001), and the perinucleus was darkly blue stained. Cell replicative senescence produced by consecutive passages treated with aspirin produced a decrease in the ratio of the percentage of SA-β-gal-positive cells both in EP BM-MSCs and LP BM-MSCs (*P* < 0.05), and the perinucleus was more lightly blue-stained than without aspirin treatment. Further, to verify aspirin itself does not affect the SA-β-gal stain, we performed negative control experiments applied passages 1 BM-MSCs (Fig. S1).

PCR analysis revealed that gene expression of senescence-associated molecules in LP BM-MSCs was down-regulated when cells were treated with aspirin (Fig. 1C). Consistent with the PCR results, western blot analysis of p21 and p53 expression levels were also reduced in cells treated with aspirin (Fig. 1D). Thus, these results indicated that aspirin can reduce cell senescence in LP BM-MSCs.

### Sequencing data of bone marrow-derived mesenchymal stromal cells

We constructed 12 cDNA libraries from four groups (samples: p5_1, p5_2, p5_3 represented for EP BM-MSCs; samples: p5_ASA_1, p5_ASA_2, p5_ASA_3 represented for EP BM-MSCs treated with aspirin; samples: p20_1, p20_2, p20_3 represented for LP BM-MSCs; samples: p20_ASA_1, p20_ASA_2, p20_ASA_3 represented for LP BM-MSCs treated with aspirin) for RNA sequencing. The main sequencing characteristics of cell specimens collected here are annotated in Table 1.

**Table 1:** RNA sequencing reads filtering and reference genome alignment.

Sample	p5_1	p5_2	p5_3	p5_ASA_1	p5_ASA_2	p5_ASA_3	p20_1	p20_2	p20_3	p20_ASA_1	p20_ASA_2	p20_ASA_3
Total Raw Reads (M)	75.15	74.86	74.87	75.15	74.86	74.87	74.53	74.9	74.74	71.13	74.65	74.53
Total Clean Reads (M)	69.1	69.1	69.16	69.1	69.1	69.16	68.38	69.13	68.84	65.02	68.58	68.38
Total Clean Bases (Gb)	6.91	6.91	6.92	6.91	6.91	6.92	6.84	6.91	6.88	6.5	6.86	6.84
Clean Reads Q20 (%)	97.88	97.8	97.83	97.88	97.8	97.83	97.89	97.84	97.88	97.84	97.89	97.89
Clean Reads Q30 (%)	92.28	91.89	92.12	92.28	91.89	92.12	92.49	92.15	92.47	92.16	92.56	92.49
Clean Reads Ratio (%)	91.95	92.3	92.38	91.95	92.3	92.38	91.75	92.3	92.11	91.4	91.86	91.75
Total Mapping (%)	95.39	95.56	95.46	95.19	95.28	95.27	95.04	95.21	95.25	95.32	95.3	94.98
Uniquely Mapping (%)	88.2	88.39	88.22	88.16	88.14	88.15	87.56	87.79	87.77	87.92	87.73	87.63

Filtering out low-quality reads, linker contamination, and reads with too high N content in unknown bases, more than 65 million clean reads were obtained in each library. Subsequently, Q30 ratio (a base quality >30 and error rate <0.001) among these clean reads was more than 91.89%. Ultimately, 94.98–95.56% clean reads were mapped to *Rattus_norvegicus* reference genome (GCF_000001895.5_Rnor_6.0).

### Identification of DEGs

**Figure 2:**
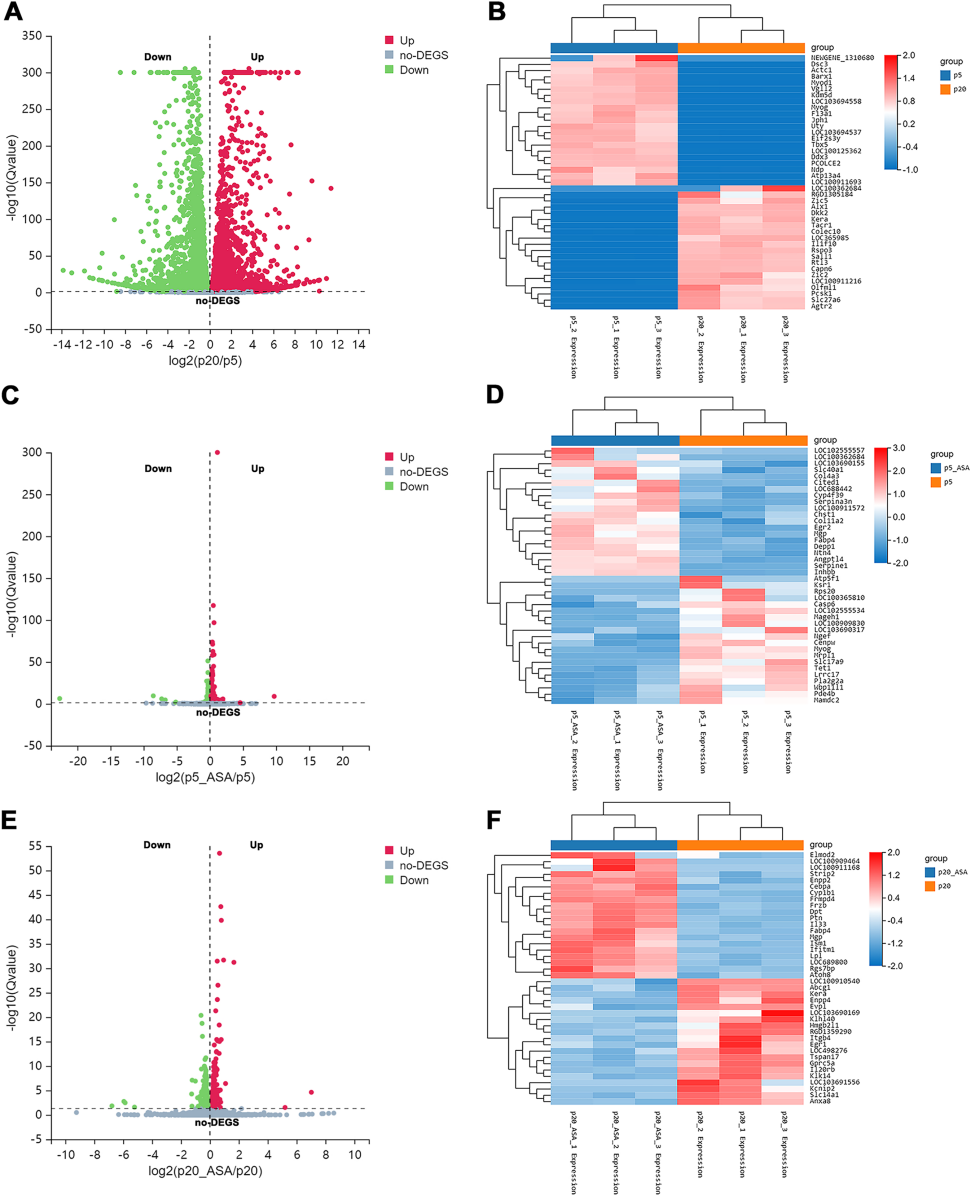
Identification of differentially expressed genes. (A) Volcano plot of EP and LP BM-MSCs. (B) Clustered heatmap of top 40 (20 up-regulated and 20 down-regulated) DEGs between the EP and LP BM-MSCs with gene annotations. (C) Volcano plot of EP BM-MSCs before and after aspirin exposure. (D) Clustered heatmap of top 40 (20 up-regulated and 20 down-regulated) DEGs between EP BM-MSCs before and after aspirin exposure with gene annotations. (E) Volcano plot of LP BM-MSCs before and after aspirin exposure. (F) Clustered heatmap of top 40 (20 up-regulated and 20 down-regulated) DEGs between LP BM-MSCs before and after aspirin exposure with gene annotations. The gradual color change from red to blue implies gene expression value from high to low; DEGs, differentially expressed genes.

After applying Bowtie2 to align clean reads to the reference gene sequence, and then using RSEM to calculate the gene expression level of each sample, we obtained differentially expressed genes (DEGs) between diverse groups *via* DESeq2 analysis (Q value <= 0.05) (Tables S1–S3). Based on this screening criterion, LP BM-MSCs compared with EP, 9,557 genes were found to be differentially expressed between these two groups (Fig. 2A). Among them, 4,632 DEGs were up-regulated in LP BM-MSCs and 4,925 DEGs were down-regulated. Further, we compared EP BM-MSCs before and after aspirin exposure, including 1,245 DEGs, of which 569 genes were up-regulated and 676 DEGs were down-regulated after aspirin exposure (Fig. 2C). Compared with LP BM-MSCs with or without aspirin treatment, including 458 DEGs, of which 228 DEGs were up-regulated and 230 DEGs were down-regulated (Fig. 2E). Additionally, clustered heatmaps of the top 20 up-regulated and down-regulated DEGs expression were shown in Figs. 2B, 2D, 2F based on z-scores normalization, respectively.

### Functional annotation and pathway enrichment analysis of DEGs

**Figure 3:**
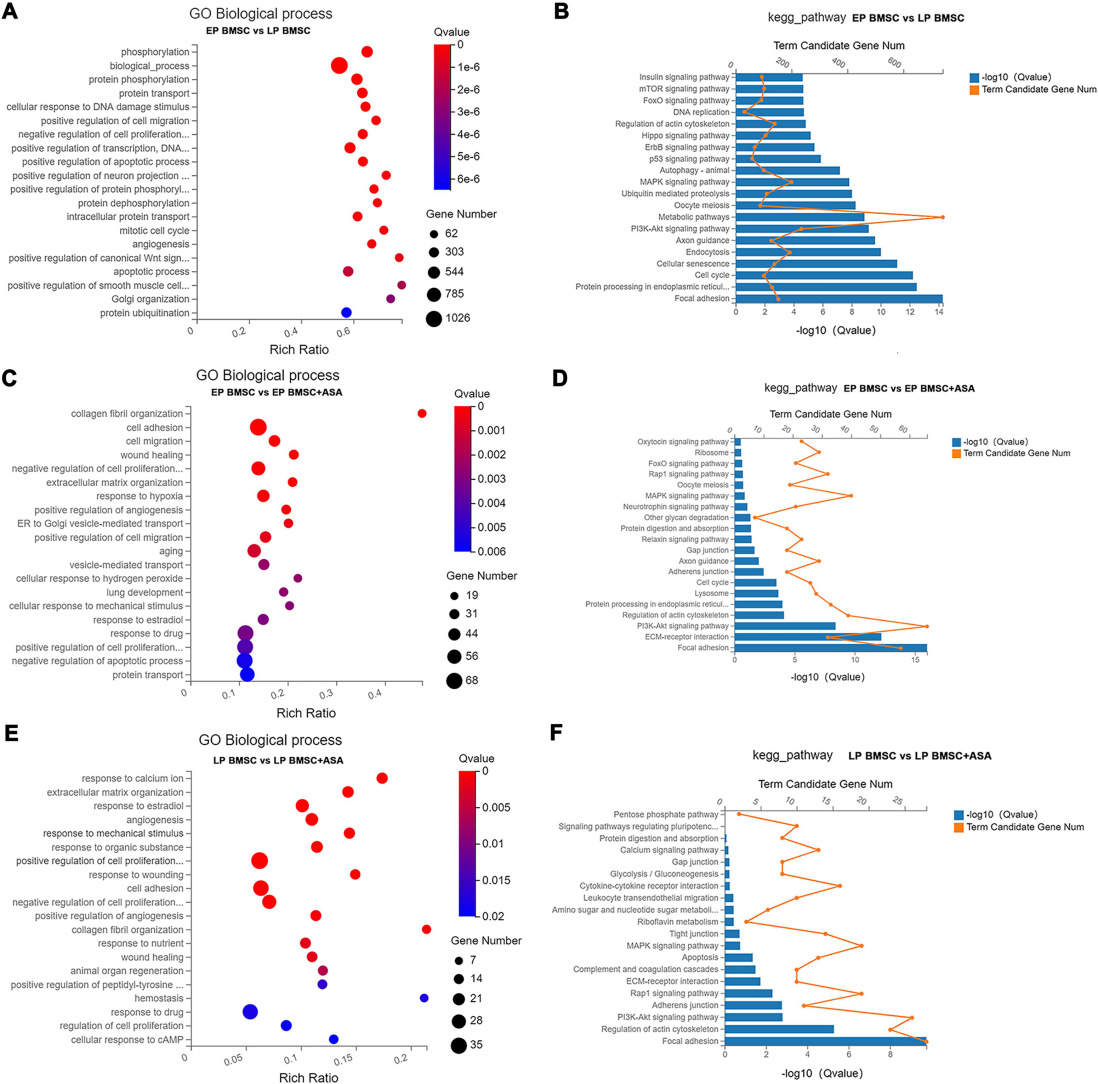
Gene Ontology (GO) and Kyoto Encyclopedia of Genes and Genomes (KEGG) pathway enrichment analysis. (A, C, E) GO enrichment analysis in different groups. The colors of circle dots illustrate the Q-values identified for each GO term (low: red, high: blue), with lower values for more significant enrichment. The size of dots indicates the number of the differentially expressed genes and the larger dots represent a larger gene number. ‘Rich Ratio’ represents the ratio of Term Candidate Gene Number to Term Gene Number. The greater the value of ‘Rich Ratio’, the more significant the enrichment. (A) GO biological process enrichment analysis of DEGs between EP and LP BM-MSCs. (C) GO biological process enrichment analysis of DEGs in EP BM-MSCs treated with aspirin. (E) GO biological process enrichment analysis of DEGs in LP BM-MSCs treated with aspirin. (B, D, F) KEGG enrichment analysis in different groups. The blue bar plots indicate −log10 Q value). The greater the value of −log10 Q value), the more significant the enrichment. The orange dots indicate the number of genes enriched to the term. (B) KEGG pathway enrichment analysis of DEGs between EP and LP BM-MSCs. (D) KEGG pathway enrichment analysis of DEGs in EP BM-MSCs treated with aspirin. (F) KEGG pathway enrichment analysis of DEGs in LP BM-MSCs treated with aspirin.

To get a sense of biological processes and pathways enriched in BM-MSCs treated with aspirin, we performed GO and KEGG pathway analysis. GO enrichment analysis results included three parts, biological processes (BP), cellular component (CC), and molecular function (MF). Detailed results of GO and KEGG enrichment analysis were summarized in Tables S4–S9. For GO analysis, the rich ratio (Rich Ratio = Term Candidate Gene Num/Term Gene Num) was plotted on the X-axis, while the GO term was plotted on the Y-axis. According to the Q value, we demonstrated the top 20 terms from small to large. Biological processes analysis of LP BM-MSCs compared with EP BM-MSCs revealed DEGs in phosphorylation, biological process, protein phosphorylation, cellular response to DNA damage stimulus, cell migration, regulation of transcription, cell cycle, apoptosis, and others (Fig. 3A). Consistently, KEGG enrichment analysis showed similar changes in cell cycle and DNA replication after BM-MSCs reached replicative senescence (Fig. 3B). Also, we observed enrichment in focal adhesion, protein processing, cellular senescence, PI3K-Akt signaling pathway, MAPK signaling pathway, p53 signaling pathway, insulin signaling pathway, and others involved in cell senescence. We next explored the biological processes that were strongly associated with EP BM-MSCs when treated with aspirin. We found cell adhesion, cell proliferation, cell migration, wound healing, response to drug, regulation of apoptotic process, protein transport and others displayed significant changes (Fig. 3C). For KEGG enrichment included focal adhesion, ECM-receptor interaction, PI3K-Akt signaling pathway, lysosome, cell cycle, and others (Fig. 3D). Inspecting LP BM-MSCs treated with aspirin through GO analysis revealed response to calcium ion, extracellular matrix organization, response to estradiol, cell adhesion, regulation of cell proliferation, and others changes enriched in this group (Fig. 3E). Focal adhesion, regulation of actin cytoskeleton, PI3K-Akt signaling pathway, adherens junction, and other KEGG pathways were enriched in LP BM-MSCs treated with aspirin (Fig. 3F). Together, these analyses indicated after serially passaged replicative senescence, DEGs between the EP BM-MSCs and LP BM-MSCs were highly enriched in the cell cycle, DNA damage stimulus, and cellular senescence (Figs. 3A–3B). After aspirin treatment, DEGs within the EP BM-MSCs and LP BM-MSCs were enriched in the focal adhesion, cell adhesion, and adherens junction pathways (Figs. 3C–3F).

### GSEA and GSVA revealed biological functions of DEGs in bone marrow-derived mesenchymal stromal cells treated with aspirin

**Figure 4:**
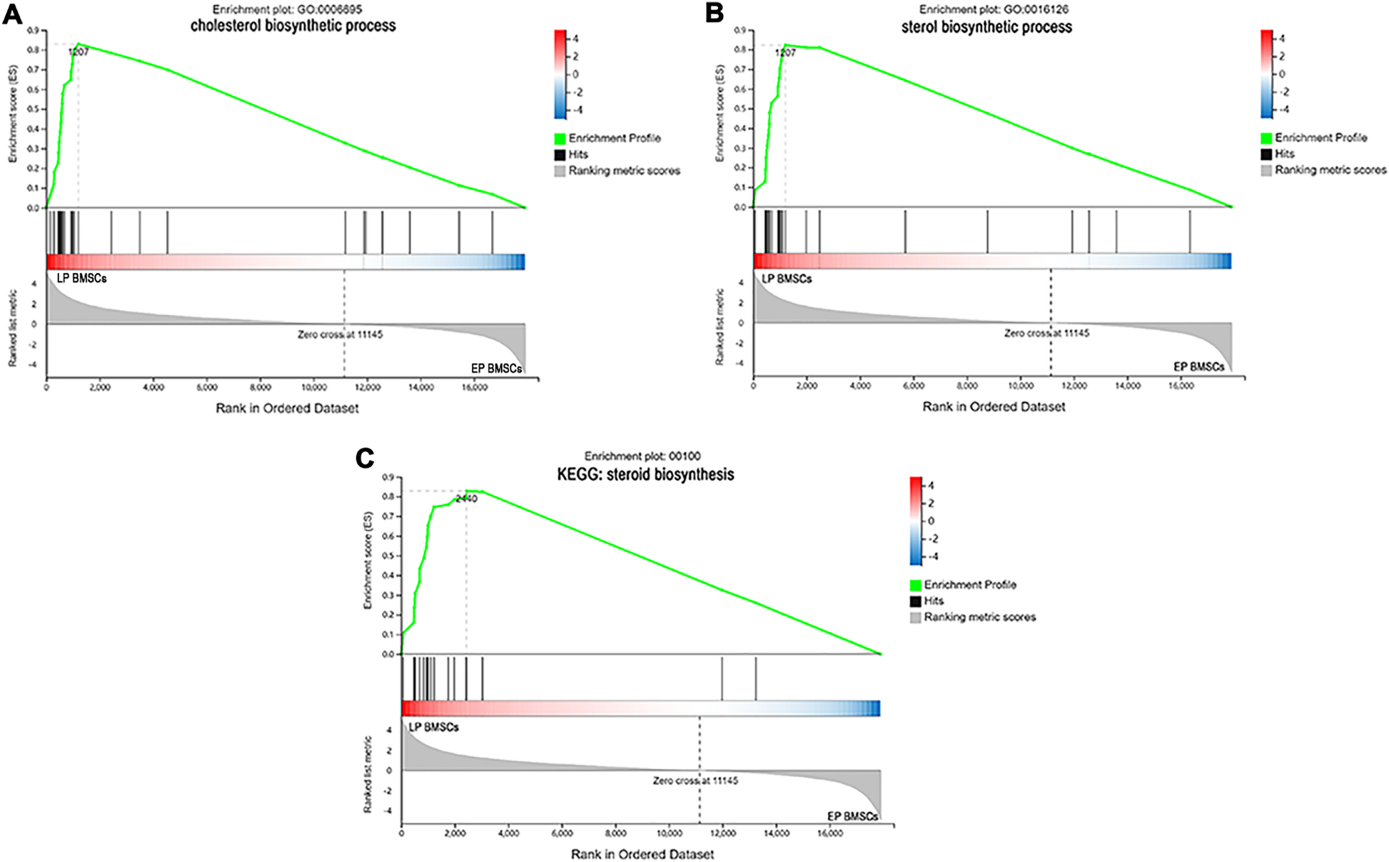
The enrichment of DEGs between EP BM-MSCs and LP BM-MSCs were analyzed *via* gene set enrichment analysis (GSEA). Mainly including cholesterol ‘biosynthetic process’ term (A), ‘sterol biosynthetic process’ term (B), and ‘steroid biosynthesis’ pathway (C). The green broken line represents the change cure of the enrichment score (ES) of the group of genes, and the Y axis is the ES value. Each black vertical line represents one gene, and this part shows all genes under this pathway or GO term. The heat map shows the signal2 noise/log2 ratio value of all expressed genes. Red means value > 0, blue means value < 0, the darker the color, the greater the absolute value.

**Figure 5:**
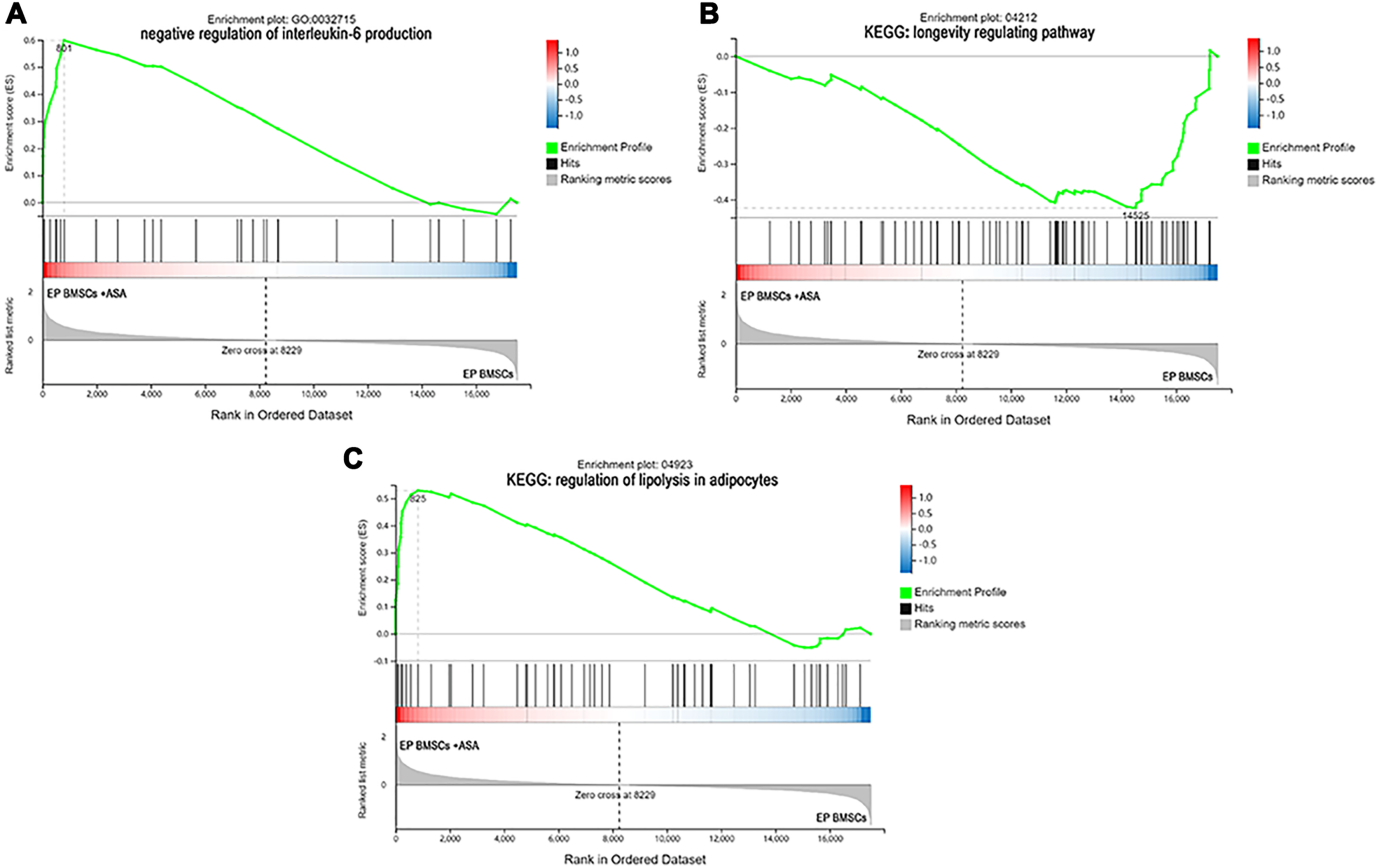
The enrichment of DEGs in EP bone marrow-derived mesenchymal stem cells treated with aspirin was analyzed *via* gene set enrichment analysis (GSEA). Mainly including ‘negative regulation of interleukin-6 production’ term (A), ‘Longevity regulating pathway’ (B), and ‘regulation of lipolysis in adipocytes’ pathway (C). The green broken line represents the change cure of the enrichment score (ES) of the group of genes, and the Y axis is the ES value. Each black vertical line represents one gene, and this part shows all genes under this pathway or GO term. The heat map shows the signal2 noise/log2 ratio value of all expressed genes. Red means value > 0, blue means value < 0, the darker the color, the greater the absolute value.

**Figure 6:**
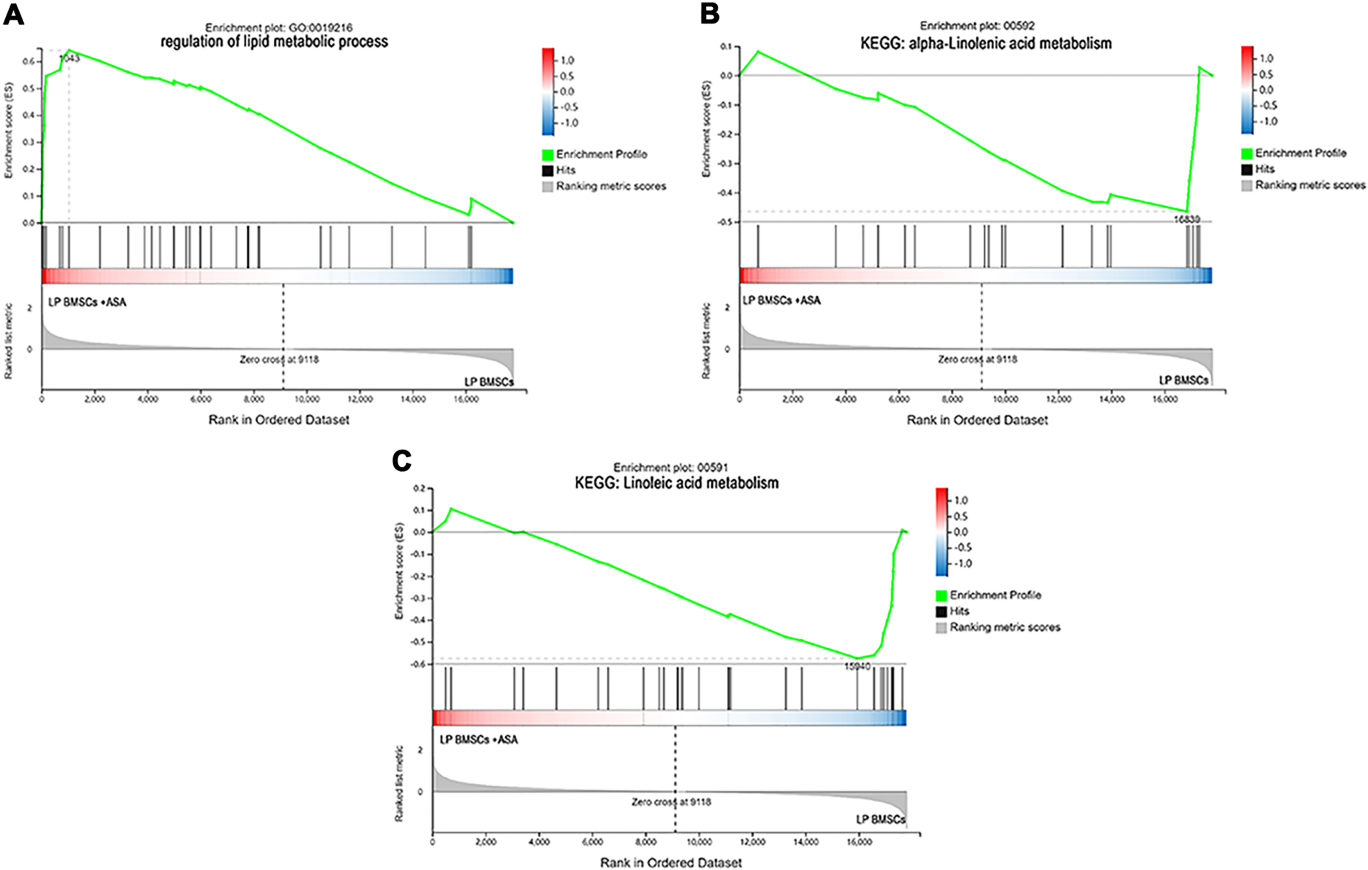
The enrichment of DEGs in LP BM-MSCs treated with aspirin was analyzed *via* gene set enrichment analysis (GSEA). Mainly including the ‘regulation of lipid metabolic process’ term (A), ‘alpha-Linolenic acid metabolism’ pathway (B), and ‘Linoleic acid metabolism’ pathway (C). The green broken line represents the change cure of the enrichment score (ES) of the group of genes, and the Y axis is the ES value. Each black vertical line represents one gene, and this part shows all genes under this pathway or GO term. The heat map shows the signal2 noise/log2 ratio value of all expressed genes. Red means value > 0, blue means value < 0, the darker the color, the greater the absolute value.

**Figure 7:**
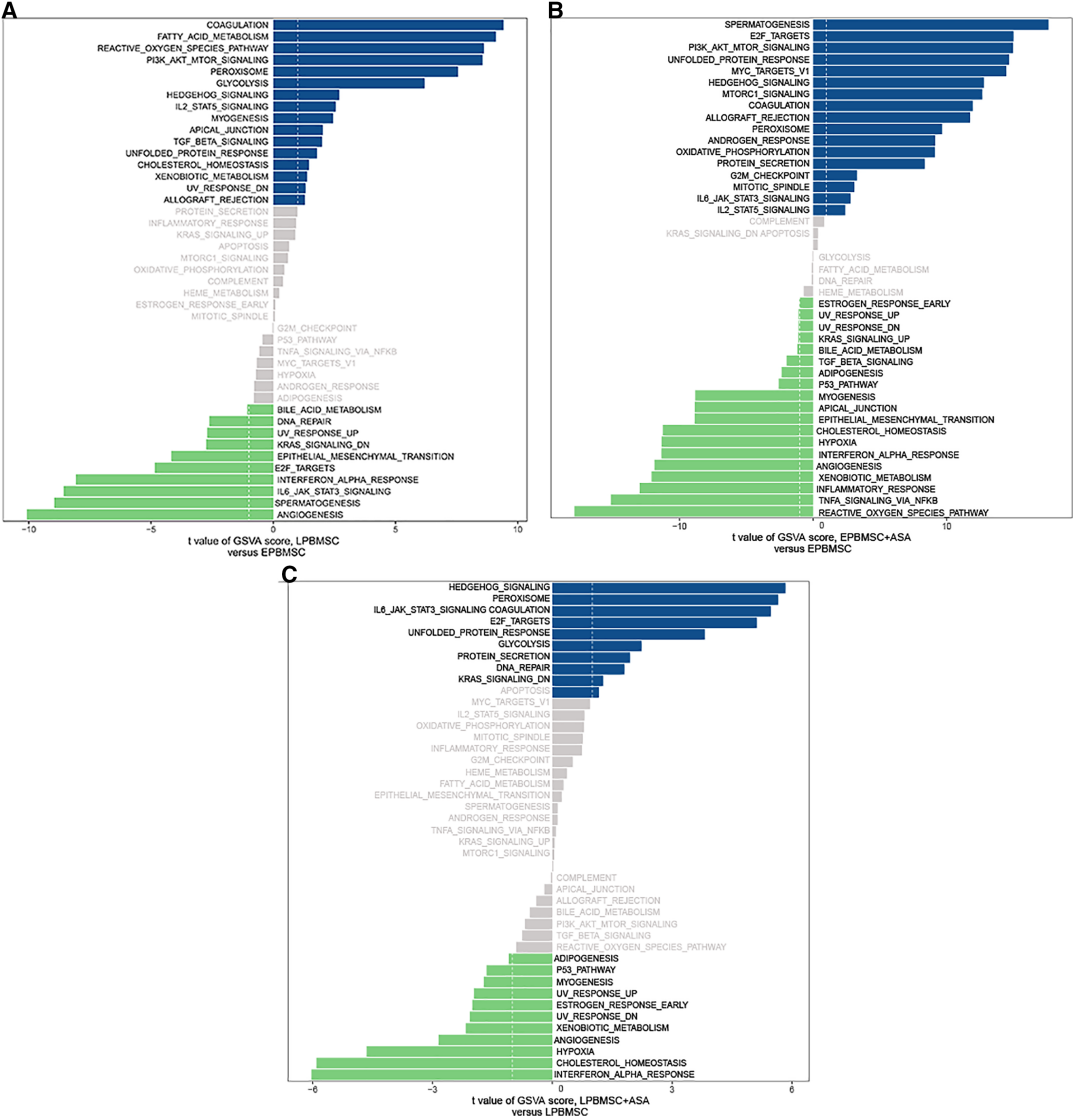
Gene set variation analysis (GSVA). (A) GSVA of differentially expressed pathways between EP BM-MSCs and LP BM-MSCs. The dark blue column shows activated pathways in the LP BM-MSCs, and the green column indicates activated pathways in the EP BM-MSCs. (B) GSVA of differentially expressed pathways in EP BM-MSCs treated with aspirin. The dark blue column shows activated pathways in the EP BM-MSCs treated with aspirin, and the green column indicates activated pathways in the EP BM-MSCs. (C) GSVA of differentially expressed pathways in LP BM-MSCs treated with aspirin. The dark blue column shows activated pathways in the LP BM-MSCs treated with aspirin, and the green column indicates activated pathways in the LP BM-MSCs. The vertical axis represents each gene set, and the horizontal axis indicates the expression difference of each gene set in different groups.

Since GO and KEGG enrichment analysis often focuses on comparing DEGs between different treatment groups, it is easy to miss some not significant yet biologically meaningful DEGs. Here, we utilized gene set enrichment analysis (GSEA), which concentrates on interpreting gene expression data by analyzing shared common biological function gene sets (Subramanian et al., 2005). GSEA of biological process and KEGG pathways detected up-regulated cholesterol biosynthetic process, sterol biosynthetic process, and steroid biosynthesis pathways in LP BM-MSCs (Figs. 4A–4C). GSEA of EP BM-MSCs treated with aspirin revealed that the addition of aspirin could down-regulated aging-related pathways such as longevity regulating pathway. After exposure to aspirin, we documented negative regulation of interleukin-6 production while regulation of lipolysis in adipocytes pathway was up-regulated (Figs. 5A–5C). Besides, GSEA implicated enrichment of several inflammation pathways and lipid-related processes in LP BM-MSCs treated with aspirin including regulation of lipid metabolic process, linoleic acid metabolism, and alpha-Linolenic acid metabolism (Figs. 6A–6C). To further verify the enrichment biological process, we profiled gene set variation analysis (GSVA) based on 50 hallmark gene sets pathways in different treatment groups. GSVA further implicated fatty acid metabolism, reactive oxygen species pathway, IL2 STAT5 signaling, and cholesterol homeostasis showed high score in LP BM-MSCs (Fig. 7A). After exposure to aspirin, the score of lipid metabolism synthesis and inflammation also decreased to varying degrees in EP and LP bone marrow-derived mesenchymal stem cells (Figs. 7B–7C).

To further validate the results of RNA-sequencing, we performed RT-PCR analysis of the DEGs and the key genes found by GSEA analysis. We provided a conclusive table of these DEGs and the key genes in GSEA analysis in Table S10 and Fig. S2.

## Discussion

Stem cells can repair damaged tissues and organs and reshape the biological functions of the body, so they are ideal seed cells for tissue engineering. Due to the limited amount of BM-MSCs obtained from the primary tissue, a substantice *in vitro* expansion is required to obtain a sufficient number of cells for clinical use. However, this process will cause the cells to undergo replicative senescence, which is also a major challenge in the current BM-MSCs research field. Previous research suggests that aspirin can impact senescence on BM-MSCs, but the mechanism has not been investigated yet. Therefore, we studied the gene expression profile changes in BM-MSCs treated with aspirin. To date, there are few studies that examine effects of aspirin’s impact on aging BM-MSCs.

Aspirin, a non-steroidal anti-inflammatory drug (NSAID), is widely used to treat inflammation, cardiovascular diseases, and senile diseases. As noted in previous studies, aspirin can extend the lifespan of *Caenorhabditis elegans* (Huang et al., 2017), *Drosophila melanogaster* (Danilov et al., 2015), *Mus musculus* (Strong et al., 2008), as well as *Homo sapiens* (Group, 2013). Moreover, emerging evidence indicates that long-term use of aspirin can improve health. In many cancer contexts, aspirin can significantly reduce the risk of many cancers, including enhancing the sensitivity to cisplatin in colorectal cancer (Jiang et al., 2020), down-regulating COX2 expression of lung cancer (Chen et al., 2019), activating AMP-activated protein kinase (AMPK) while inhibiting mTORC1 signaling in breast cancer (Henry et al., 2017), and other different mechanisms. In type 2 diabetes, high-dose aspirin promotes glucose metabolism and reduces fatty acid levels (Hundal et al., 2002). Besides, aspirin has considerable curative effects on neurodegenerative diseases, such as Parkinson’s disease (Ren et al., 2018), and Alzheimer’s disease (Group et al., 2008). Therefore, it is desirable to speculate that aspirin has anti-aging properties. A previous study explained that aspirin retards senescence of endothelial cells by reducing reactive oxygen species (ROS), increasing nitric oxide (NO) and cGMP levels (Bode-Boger et al., 2005). Cellular senescence in human and mouse fibroblasts was suppressed by aspirin *via* inhibiting COX2 expression (Feng et al., 2019). Together with our finding that aspirin could retard senescence in different species, including *Homo sapiens*, *Mus musculus*, and *Rattus norvegicus*. The difference between previous studies and our study was not only the species of experiment animal cell but also the concentration of aspirin. We applied 400 μmol/L aspirin, which was higher than the above-mentioned studies. In rat BM-MSCs, this dosage didn’t affect apoptosis and proliferation (Zhan et al., 2018). Hence, within different types of cells, aspirin could retard senescence to a certain extent with diverse concentrations. Besides, our results also showed aspirin could influence immune response and lipid metabolism in cells.

In this study, we present a systematic comparative analysis of aspirin’s impact on EP and LP BM-MSCs. By using an original experiment and RNA sequencing approach, a comprehensive aspirin-BM-MSCs network for all differentially expressed genes was generated. Further, we performed GO functional enrichment and KEGG pathway analysis, GSEA, and GSVA to facilitate comparative studies in EP and LP BM-MSCs. Herein, this study offers new insights for alleviating the effects of aspirin on the senescence of BM-MSCs, and may open up the possibility of the application of aspirin in treating aging-related diseases.

To analyze whether aspirin could slow cellular senescence, we conducted conventional measurements of aging, including gene and protein expression levels of p16, p21, p53, and SA β-gal staining. Cyclin-dependent kinase inhibitor 2a (CDKN2A/p16Ink4a) expression has been identified as a superior biomarker of aging (Hudgins et al., 2018; Krishnamurthy et al., 2004). Direct evidence supporting a role for p16Ink4a in rat BM-MSCs has been widely applied, especially the increased expression in senescent BM-MSCs (Yusop et al., 2018; Zhang et al., 2015; Zheng et al., 2016). Further, cyclin-dependent kinase inhibitor p21 (CDKN1A) can act as a key molecular mediator of therapy-induced senescence (Abbas & Dutta, 2009; Cazzalini et al., 2010). P21 halts the cell-cycle progression after transcriptional activation by p53, which is a DNA damage response triggered by many senescence-inducing agents. The role of p21 in senescent cells has also been confirmed in rat BM-MSCs (Wang et al., 2020; Yang et al., 2020; Zhang et al., 2016). Typically, p53 is listed as an indicator of aging along with p16 and p21. Activation of p53 is involved in various processes including cell cycle arrest, apoptotic cell death, and cellular senescence (Li et al., 2012; Schmitt et al., 2002; Vousden & Prives, 2009). Similarly, up-regulation of p53 is a consistent feature of cellular senescence in rat BM-MSCs (Yang et al., 2020; Zhang et al., 2018; Zheng et al., 2016). Our results suggested that adding aspirin to EP BM-MSCs and LP BM-MSCs could decrease aging indicators (Fig. 1). Subsequently, we performed RNA-seq to explore the potential mechanism of aspirin’s impact on BM-MSCs. Biological process and KEGG enrichment analysis indicated that several pathways related to cellular processes were significantly enriched in LP BM-MSCs compared with EP BM-MSCs, including cellular senescence and p53 signaling pathways (Figs. 3A–3B). This suggested that LP BM-MSCs were senescent. Further, GSEA and GSVA analysis of biological process and KEGG pathways identified cholesterol, sterol, steroid, and other lipids biosynthetic as significantly increased in LP BM-MSCs compared with EP BM-MSCs (Figs. 4A–4C, 7A). Consistent with our findings, emerging evidence indicates the accumulation of lipids in aging cells including BM-MSCs (Lu et al., 2019; Seo et al., 2019; Stolzing & Scutt, 2006). BM-MSCs underwent excessive adipogenic differentiation rather than osteogenic differentiation, which resulted in bone metabolism imbalanced and bone mass losing, during the abuse of hormones, menopause, and aging (Liu, Xia & Li, 2015). Consistently, a trial conducted on bone marrow-derived mesenchymal stem cells, indicated that global lipid distribution and the relevant metabolic flows were disturbed when bone marrow-derived mesenchymal stem cells after serially passaging (Lu et al., 2019).

For EP BM-MSCs treated with aspirin, analysis of biological process and KEGG enrichment, excluding broad pathways, found several pathways involved senescence, immune response, and lipid metabolism. For example, they included focal adhesion, ECM-receptor interaction, PI3K-Akt signaling pathway, cell cycle, protein digestion and absorption, MAPK signaling pathway, FoxO signaling pathway, and others (Figs. 3C–3D). Among these, GSEA analysis showed the DEGs were significant with negative regulation of interleukin-6 (IL-6) production, longevity regulating pathway, and regulation of lipolysis in adipocytes (Figs. 5A–5C). On the other hand, we found reactive oxygen species (ROS) pathway, inflammatory response retained the lowest t value of GSVA score in GSVA analysis (Fig. 7B). These findings suggested that aspirin might influence the immune response and lipid metabolism on EP BM-MSCs to slow cellular senescence. As noted in previous studies, the secretion of IL-6 is a characteristic of the secretion of senescence-associated inflammatory cytokines (SASP), which is promoted by persistent DNA damage (Rodier et al., 2009). Also, one common denominator of aging is increasing ROS production resulted from progressive mitochondrial dysfunction (Harman, 1965). Aspirin has previously been shown to diminish ROS and inflammatory cytokines, including IL-1β, IL-6, and tumor necrosis factor-alpha (TNF-α) (Liu et al., 2019; Wang, Huang & Zuo, 2014). Regarding aspirin’s influence on lipid metabolism, we took note of some previous reports showing aspirin could inhibit the adipogenic differentiation of BM-MSCs and preadipocytes (Su et al., 2014; Zhan et al., 2018). Although this observation was not verified in cells not induced to adipogenesis. Combined with our findings, aspirin might alleviate the accumulation of lipids in cells, reduce the accumulation of intracellular ROS, and eliminate inflammatory cytokines to retard aging in EP BM-MSCs.

For LP BM-MSCs treated with aspirin, the analysis of biological process and KEGG enrichment were consistent with EP BM-MSCs treated with aspirin, and included several pathways involved in senescence, including focal adhesion, PI3K-Akt signaling pathway, ECM-receptor interaction, apoptosis, MAPK signaling pathway, cytokine-cytokine receptor interaction, protein digestion and absorption, and other pathways (Figs. 3E–3F). GSEA analysis showed the DEGs were significant concerning linoleic acid metabolism, alpha-linolenic acid metabolism, and regulation of lipid metabolic process (Figs. 6A–6C). On the other hand, GSVA analysis uncovered interferon α response retained the lowest t value of GSVA (Fig. 7C). These observations, consistent with EP BM-MSCs treated with aspirin, illustrated that aspirin might influence lipid metabolism and impact immune response. It was also possible that linoleic acid caused senescence through multiple mechanisms, as previous studies demonstrate increasing IKKβ activity and decreasing autophagic flux (Kim et al., 2005; Lee et al., 2015; Raederstorff, Loechleiter & Moser, 1995). In our study, we found linolenic acid metabolism was downregulated by the addition of aspirin in LP BM-MSCs, in agreement with previous observations in human serum (Ellero-Simatos et al., 2015). In addition, interferon response can contribute to aging, as exemplified by studies with senescent rats in multiple tissues (O’Brown et al., 2015; Shavlakadze et al., 2019). The administration of aspirin was capable of inhibiting IFN-γ production (Cao et al., 2015; Liu et al., 2011), which indicated aspirin might be responsible for delaying senescence by inhibiting interferon response.

Our study showed that aspirin could delay BM-MSCs senescence to a certain extent. The mechanisms of this delay might involve influencing immune response and lipid metabolism. These findings may allow better understanding and clinical application of aspirin-treated BM-MSCs. In this study, we performed an original experiment and RNA-seq to detect aspirin’s influence on bone marrow-derived mesenchymal stem cells, this research still existed restrictions. Initially, we chose a concentration of 400 μmol/L aspirin because it did not affect cell proliferation and apoptosis. However, this dosage is still greater than the blood concentration in the human body after oral administration of aspirin (Hobl et al., 2015; Levy, 1978; Lucker et al., 1992). Therefore, if aspirin is applied to the clinic to delay aging, it is also necessary to increase the long-term use of low-concentration aspirin *in vivo*/*in vitro* experimental research. Second, due to the differential biological process and KEGG pathways both being involved in promoting senescence and delaying senescence, combined with our experiment finding, we only focused on inhibiting senescence-related pathways. This led to the analysis of the effect of aspirin on BM-MSCs that didn’t reach sufficient depth. Third, our previous research showed aspirin could interfere with adipogenic differentiation of BM-MSCs by inhibiting HDAC9 expression. In the present study, we found the potential mechanisms of aspirin delayed cell senescence included inhibiting lipid accumulation. As predicted, HDAC9 expression was elevated in aging BM-MSCs (Fig. S3A). After treatment with aspirin, HDAC9 expression was down-regulated (Fig. S3B). This likely indicates HDAC9 participates in the senescent process and is regulated aspirin. However, the exact role of HDAC9 in the aging process has not been verified. Further studies are needed to define these intricate mechanisms. Fourth, although aspirin was found to retard BM-MSCs senescence, it has not been verified in other cell types. Fifth, in our study, we have verified the meaningful genes by performing PCR experiment. Silencing and overexpression experiments of these meaningful genes will be necessary to verify the function in the future. Whether aspirin and its products could retard cell senescence and be applied locally in the clinical setting is an important topic for future studies.

## Conclusion

In summary, aspirin could delay BM-MSCs senescence. The potential mechanisms include influencing immune response and lipid metabolism.

## Supplemental Information

10.7717/peerj.12819/supp-1 Supplemental Information 1 Senescence-associated β-galactosidase (SA-β-gal) staining of bone marrow-derived mesenchymal stem cells at passage 1. Senescence-associated β-galactosidase (SA-β-gal) staining of bone marrow-derived mesenchymal stem cells at passage 1 with or without aspirin treatment (400 μmol/L). Scale bar = 100 μm.

10.7717/peerj.12819/supp-2 Supplemental Information 2 Validation of key genes after aspirin treatment based on GSEA analysis. Gene expression of Scd, Sirt1, and Pla2g2a in different groups. *P < 0.05, *P < 0.01, ****P < 0.0001, NS: no significance, with comparisons indicated by lines.

10.7717/peerj.12819/supp-3 Supplemental Information 3 Validation of Hdac9 gene expression after aspirin treatment. Gene expression of Hdac9 in different groups. *P < 0.05, *P < 0.01, with comparisons indicated by lines.

10.7717/peerj.12819/supp-4 Supplemental Information 4 Different expression genes (DEGs) between p5 and p20.

10.7717/peerj.12819/supp-5 Supplemental Information 5 Different expression genes (DEGs) between p5 and p5 treated with aspirin.

10.7717/peerj.12819/supp-6 Supplemental Information 6 Different expression genes (DEGs) between p20 and p20 treated with aspirin.

10.7717/peerj.12819/supp-7 Supplemental Information 7 GO analysis of different expression genes (DEGs) between p5 and p5 treated with aspirin.

10.7717/peerj.12819/supp-8 Supplemental Information 8 GO analysis of different expression genes (DEGs) between p5 and p20 treated with aspirin.

10.7717/peerj.12819/supp-9 Supplemental Information 9 GO analysis of different expression genes (DEGs) between p20 and p20 treated with aspirin.

10.7717/peerj.12819/supp-10 Supplemental Information 10 KEGG analysis of different expression genes (DEGs) between p5 and p20.

10.7717/peerj.12819/supp-11 Supplemental Information 11 KEGG analysis of different expression genes (DEGs) between p5 and p5 treated with aspirin.

10.7717/peerj.12819/supp-12 Supplemental Information 12 KEGG analysis of different expression genes (DEGs) between p20 and p20 treated with aspirin.

10.7717/peerj.12819/supp-13 Supplemental Information 13 Genes of interest and associated functions of interest arising after aspirin treatment in bone marrow-derived mesenchymal stem cells *in vitro* culture. Log2FC represents log2 fold change. Positive number of log2FC(p20/p5) is upregulated and negative is downregulated *versus* P5. Positive number of log2FC(p5+A/p5) is upregulated and negative is downregulated *versus* P5. Positive number of log2FC(p20+A/p20) is upregulated and negative is downregulated *versus* P20.

10.7717/peerj.12819/supp-14 Supplemental Information 14 Raw data for Fig. 1B.

10.7717/peerj.12819/supp-15 Supplemental Information 15 Raw data for Fig. 1C.

10.7717/peerj.12819/supp-16 Supplemental Information 16 Uncropped blots of p21 in Fig. 1C.

10.7717/peerj.12819/supp-17 Supplemental Information 17 Uncropped blots of p53 in Fig. 1C.

10.7717/peerj.12819/supp-18 Supplemental Information 18 Uncropped blots of GAPDH in Fig. 1C.

10.7717/peerj.12819/supp-19 Supplemental Information 19 DEGs between p5+ASA and p20+ASA.

10.7717/peerj.12819/supp-20 Supplemental Information 20 DEGs between p5 and p5+ASA.

10.7717/peerj.12819/supp-21 Supplemental Information 21 DEGs between p5 and p20.

10.7717/peerj.12819/supp-22 Supplemental Information 22 DEGs between p20 and p20+ASA.

10.7717/peerj.12819/supp-23 Supplemental Information 23 Raw data for Figures S2 and S3.

10.7717/peerj.12819/supp-24 Supplemental Information 24 Primer sequences of validated genes in Figures S2 and S3.

## List of abbreviations

COX I: cyclooxygenase I
COX II: cyclooxygenase II
EP bone marrow-derived mesenchymal stem cells: early passages bone marrow-derived mesenchymal stem cells
LP bone marrow-derived mesenchymal stem cells: late passages bone marrow-derived mesenchymal stem cells
DEGs: different expression genes
BP: biological processes
CC: cellular component
MF: molecular function
GSEA: gene set enrichment analysis
GSVA: gene set variation analysis
NSAID: non-steroidal anti-inflammatory drug
AMPK: AMP-activated protein kinase
ROS: reactive oxygen species
NO: nitric oxide
IL-6: interleukin-6
SASP: senescence-associated inflammatory cytokines
TNF-α: tumor necrosis factor-alpha
PPI: protein-protein interaction

## Additional Information and Declarations

### DNA Deposition

The following information was supplied regarding the deposition of DNA sequences: The raw data is available at SRA: PRJNA731238.

### Data Availability

The following information was supplied regarding data availability: The sequences data described here were uploaded in Supplemental Files.
