# Supplementary figures and images for: Characterization of transcriptional landscape in bone marrow-derived mesenchymal stromal cells treated with aspirin by RNA-seq
Source: PeerJ. 2022 Jan 24;10:e12819. doi: 10.7717/peerj.12819 (PMC8793730; doi:10.7717/peerj.12819)

Aspirin (400  $\mu\text{mol/L}$ )

—

+

SA- $\beta$ -Gal

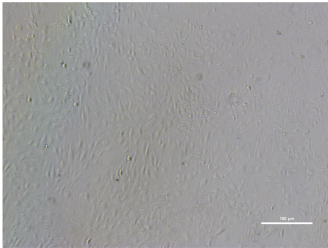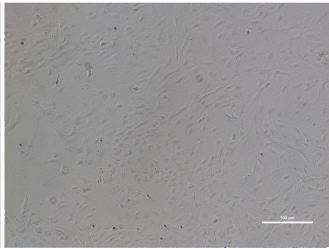

Supplement: Supplemental Information 1 — Senescence-associated β-galactosidase (SA-β-gal) staining of bone marrow-derived mesenchymal stem cells at passage 1 with or without aspirin treatment (400 μmol/L). Scale bar = 100 μm. [file peerj-10-12819-s001.pdf]

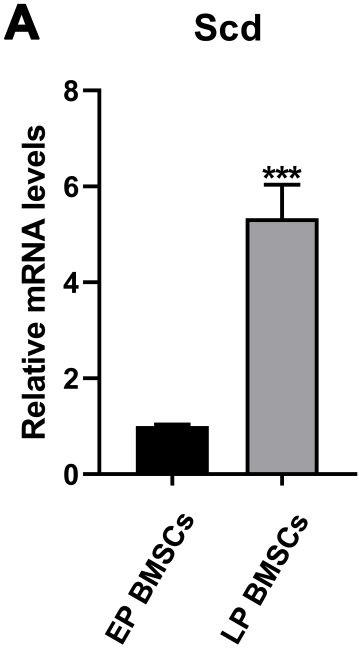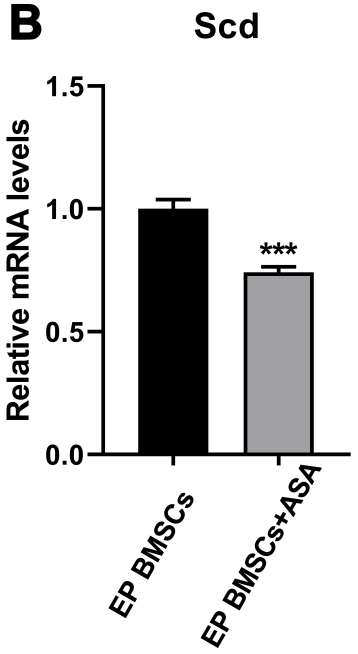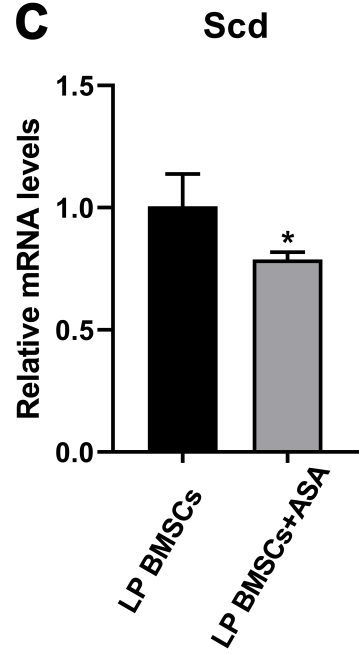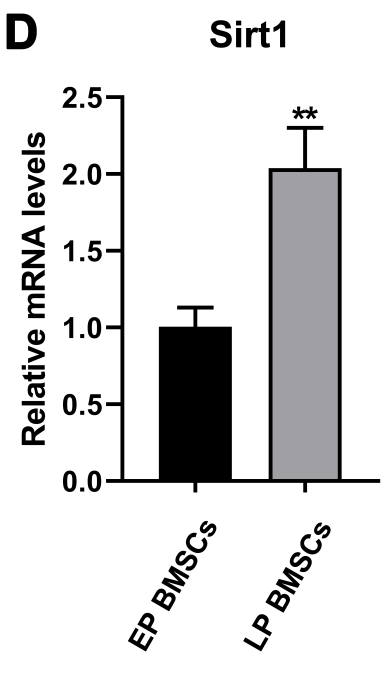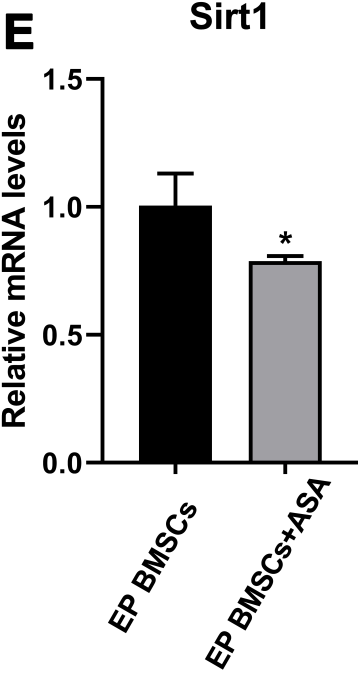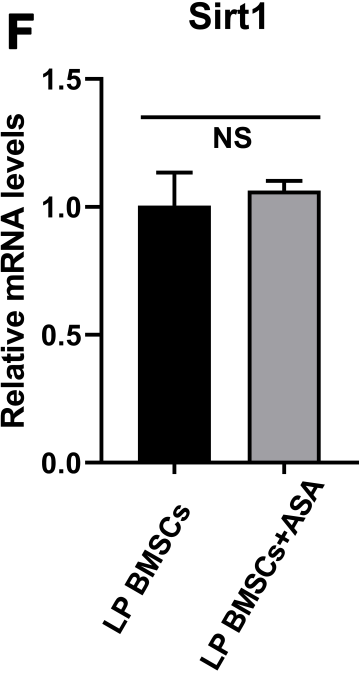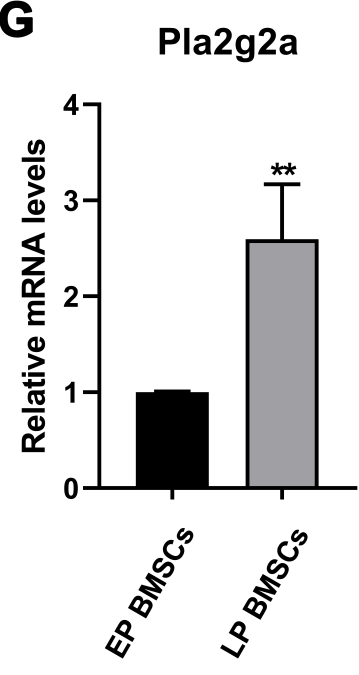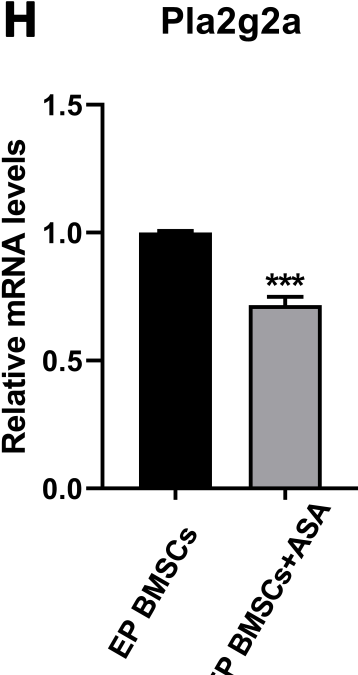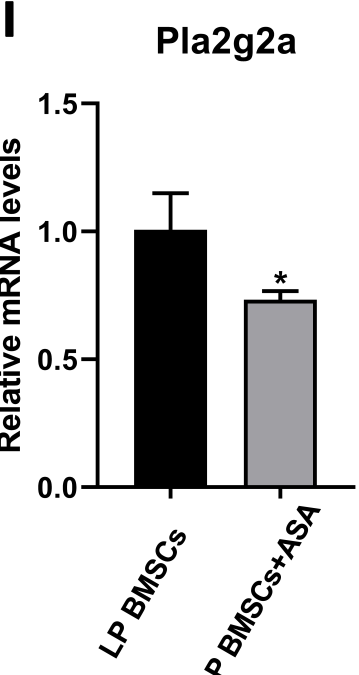

Supplement: Supplemental Information 2 — Gene expression of Scd, Sirt1, and Pla2g2a in different groups. *P < 0.05, *P < 0.01, ****P < 0.0001, NS: no significance, with comparisons indicated by lines. [file peerj-10-12819-s002.pdf]

**A****Hdac 9**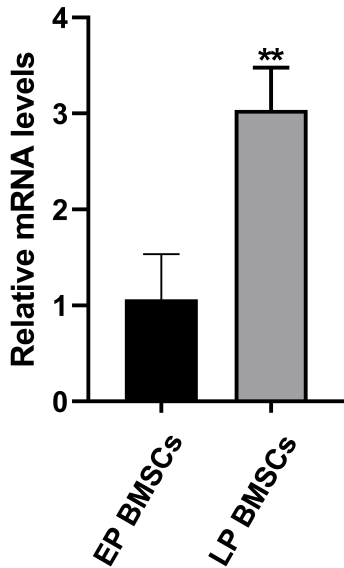**B****Hdac 9**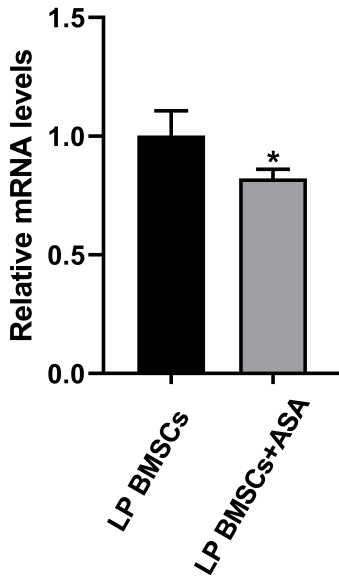

Supplement: Supplemental Information 3 — Gene expression of Hdac9 in different groups. *P < 0.05, *P < 0.01, with comparisons indicated by lines. [file peerj-10-12819-s003.pdf]

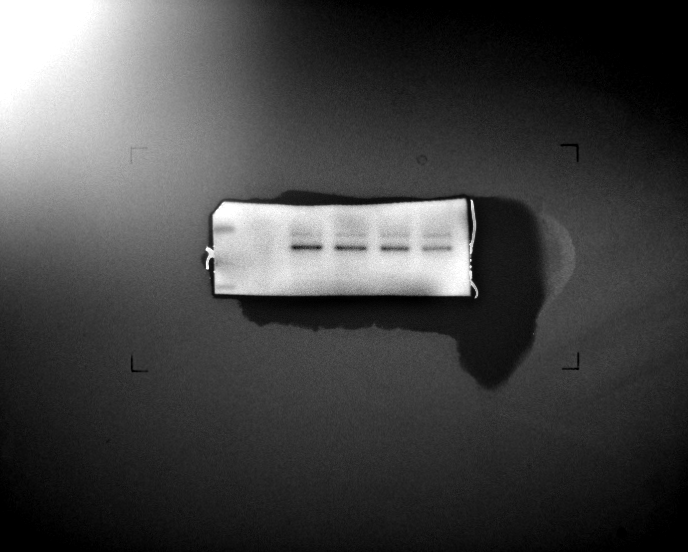

Supplement: Supplemental Information 16 [file peerj-10-12819-s016.tif]

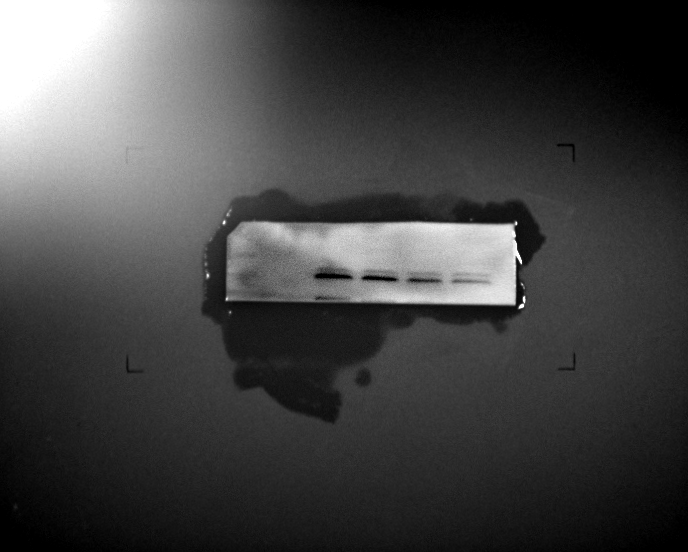

Supplement: Supplemental Information 17 [file peerj-10-12819-s017.tif]

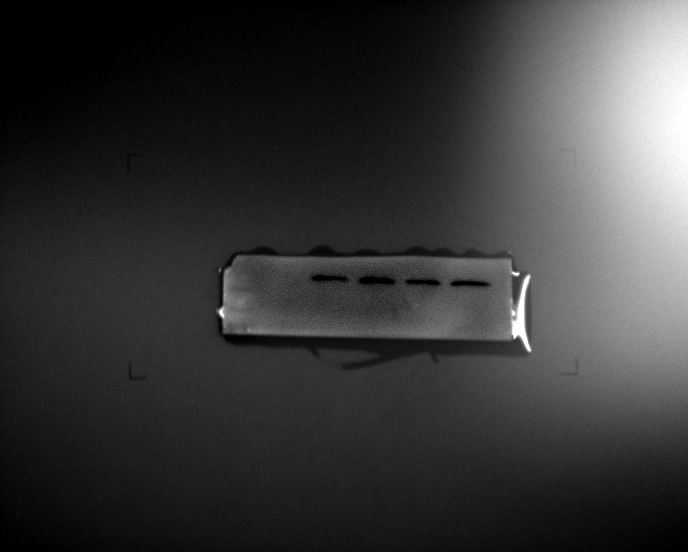

Supplement: Supplemental Information 18 [file peerj-10-12819-s018.tif]
