# Supplementary material for: Characterization of transcriptional landscape in bone marrow-derived mesenchymal stromal cells treated with aspirin by RNA-seq
Source: PeerJ. 2022 Jan 24;10:e12819. doi: 10.7717/peerj.12819 (PMC8793730; doi:10.7717/peerj.12819)
Supplement: Supplemental Information 13 — Log2FC represents log2 fold change. Positive number of log2FC(p20/p5) is upregulated and negative is downregulated versus P5. Positive number of log2FC(p5+A/p5) is upregulated and negative is downregulated versus P5. Positive number of log2FC(p20+A/p20) is upregulated and negative is downregulated versus P20. [file peerj-10-12819-s013.docx]

Supplementary Table S10. Genes of interest and associated functions of interest arising after aspirin treatment in bone marrow-derived mesenchymal stem cells in vitro culture.

| Early passages vs late passages bone marrow-derived mesenchymal stem cells | | |
| --- | --- | --- |
| Symbol | Log2FC(p20/p5) | Function |
| Apoe | 3.57 | KEGG: Cholesterol metabolism |
| Tm7sf2 | 4.53 | KEGG: Lipid metabolism |
| Cyp51 | 2.05 | GO: lipid metabolic process |
| Lss | 1.68 | GO: lipid metabolic process |
| Early passages bone marrow-derived mesenchymal stem cells treated with or without aspirin. | | |
| Symbol | Log2FC(p5+A/p5) | Function |
| Sirt1 | -0.23 | Aging |
| Scd | -0.44 | Lipid metabolism |
| Late passages bone marrow-derived mesenchymal stem cells treated with or without aspirin. | | |
| Symbol | Log2FC(p5+A/p5) | Function |
| Pla2g2a | -0.38 | KEGG: Linoleic acid metabolism |
| Scd | -0.39 | Lipid metabolism |
